# Detection of Dominant Intra-prostatic Lesions in Patients With Prostate Cancer Using an Artificial Neural Network and MR Multi-modal Radiomics Analysis

**DOI:** 10.3389/fonc.2019.01313

**Published:** 2019-11-26

**Authors:** Hassan Bagher-Ebadian, Branislava Janic, Chang Liu, Milan Pantelic, David Hearshen, Mohamed Elshaikh, Benjamin Movsas, Indrin J. Chetty, Ning Wen

**Affiliations:** ^1^Department of Radiation Oncology, Henry Ford Health System, Detroit, MI, United States; ^2^Department of Radiology, Henry Ford Health System, Detroit, MI, United States

**Keywords:** radiomics, multiparametric MRI (mpMRI), prostate cancer, intraprostatic lesion, artifical neural network (ANN)

## Abstract

**Purpose:** The aim of this study was to identify and rank discriminant radiomics features extracted from MR multi-modal images to construct an adaptive model for characterization of Dominant Intra-prostatic Lesions (DILs) from normal prostatic gland tissues (NT).

**Methods and Materials:** Two cohorts were retrospectively studied: Group A consisted of 98 patients and Group B 19 patients. Two image modalities were acquired using a 3.0T MR scanner: Axial T2 Weighted (T2W) and axial diffusion weighted (DW) imaging. A linear regression method was used to construct apparent diffusion coefficient (ADC) maps from DW images. DILs and the NT in the mirrored location were drawn on each modality. One hundred and sixty-eight radiomics features were extracted from DILs and NT. A Partial-Least-Squares-Correlation (PLSC) with one-way ANOVA along with bootstrapping ratio techniques were recruited to identify and rank the most discriminant latent variables. An artificial neural network (ANN) was constructed based on the optimal latent variable feature to classify the DILs and NTs. Nineteen patients were randomly chosen to test the contour variability effect on the radiomics analysis and the performance of the ANN. Finally, the trained ANN and a two dimension (2D) convolutional sampling method were combined and used to estimate DIL-NT probability map for two test cases.

**Results:** Among 168 radiomics-based latent variables, only the first four variables of each modality in the PLSC space were found to be significantly different between the DILs and NTs. Area Under Receiver Operating Characteristic (AUROC), Positive Predictive and Negative Predictive values (PPV and NPV) for the conventional method were 94%, 0.95, and 0.92, respectively. When the feature vector was randomly permuted 10,000 times, a very strong permutation-invariant efficiency (*p* < 0.0001) was achieved. The radiomic-based latent variables of the NTs and DILs showed no statistically significant differences (F_statistic_ < Fc = 4.11 with Confidence Level of 95% for all 8 variables) against contour variability. Dice coefficients between DIL-NT probability map and physician contours for the two test cases were 0.82 and 0.71, respectively.

**Conclusion:** This study demonstrates the high performance of combining radiomics information extracted from multimodal MR information such as T2WI and ADC maps, and adaptive models to detect DILs in patients with PCa.

## Introduction

Radiation Therapy (RT) has been proven to be an effective form of treatment for prostate cancer (PCa) and still is considered as one of the standard treatment options available. The current practice is to treat the entire prostate with a homogeneous dose distribution (1, 2). Escalated dose conformal radiotherapy has shown an advantage in biochemical progression-free survival but it is associated with the increase in acute and late toxicities (3). Simultaneous dose escalation to the dominant intra-prostatic lesions (DILs), while maintaining acceptable doses to the whole prostate gland has potential to improve therapeutic ratio for prostate cancer patients. A median dose to the entire gland could prevent the disease recurrence in the prostate from satellite tumors and significantly reduce the side effects associated with escalated radiation dose to the entire gland. A boosting dose to the DIL can maintain the effectiveness of focal therapy to treat the DIL that is the main determinant for tumor progression and prognosis. For this strategy to be successful, key requirements are the ability to accurately and reliably identify clinically significant tumors in the prostate gland.

Among different imaging techniques, Magnetic Resonance Imaging (MRI) is used increasingly and provides clinicians and researchers with useful information for delineation of the prostate gland and clinically significant tumors in PCa patients (1, 2, 4). While multi-parametric (MP) MRI is well-established (5, 6) for detection of lesions and for staging of the disease, the sensitivity for small and lower grade lesions as well as spare tumors has been low (7) and MP-MRI has failed to improve the detection accuracy of lesions in the central gland (8). Furthermore, accurate and automatic delineation of DILs from prostate glandular tissue which is not a common practice, still remains a challenge. Radiomics analysis, which is defined as the post-processing for high throughput extraction of textural and intensity-based information from medical images, can play a central role toward detecting biomarkers for diagnosis and/or therapy of patients with cancer (9, 10).

This study aims to identify discriminant radiomics features in the real radiomics-feature space and the latent-variable space (constructed from radiomics features in the space of Partial Least Square Correlation, PLSC) for construction of an adaptive model to classify DILs and NTs. The discriminant feature set in the PLSC latent-variable space can also be used for intra-tumoral segmentation and treatment response evaluation.

## Methods and Materials

### Patient Population, and Pre-processing

A total of hundred-seventeen patients consisted of the following two groups were studied:

***Group A:*** This group consisted of 98 PCa patients collected in Radboud University Nijmegen Medical Centre (11) and evaluated with Computer-Aided Diagnosis (CAD) (12, 13). Each MR study was read and reported by or under the supervision of an expert radiologist (Barentsz), with more than 20 years of experience in prostate MR. The radiologist indicated areas of suspicion with a score per modality using a point marker. If an area was considered likely for cancer a biopsy was performed. All biopsies were performed under MR-guidance and confirmation scans of the biopsy needle *in situ* were made to confirm accurate localization. Biopsy specimen were subsequently graded by a pathologist and the results were used as ground truth. Gleason grade groups for these patients are listed in Table 1, GroupA.

**Table 1:** Gleason Grade Group and PSA level of PCa patients for the two groups are shown in the table.

	**Gleason grade group**	**No. of patients**
**GROUP A**		
	1	30
	2	39
	3	19
	4	5
	5	5
**GROUP B**		
	1	5
	2	7
	3	3
	4-5	4
	**PSA level**	**No. of patients**
	<4	5
	4-10	8
	10–20	2
	>20	4

*The PSA levels are not available for group A*.

All MR studies included T2-weighted (T2W) and diffusion-weighted (DW) imaging. The images were acquired on two different types of Siemens 3T MR scanners, the MAGNETOM Trio and Skyra. T2W images were acquired using a turbo spin echo sequence and had a resolution of around 0.5 mm in plane and a slice thickness of 3.6 mm. the DWI series were acquired with a single-shot echo planar imaging sequence with a resolution of 2 mm in-plane and 3.6 mm slice thickness and with diffusion-encoding gradients in three directions. Three b-values were acquired [50, 400, and 800 (sec-mm^−2^)], and subsequently, the ADC map was calculated by the scanner software. All images were acquired without an endorectal coil, as per the PI-RADS guidelines for acquisition of prostate MRI (14).

***Group B:*** Consisted of 19 patients (age range: 56–84, mean: 67) collected in our hospital, presented with increased PSA levels, suspicion in MR images, and biopsy-proven localized prostate carcinoma with no prior treatment. PSA and Gleason score of these patients are listed in Table 1, GroupB. All patients underwent an MP MRI study. An ultrasound guided needle biopsy was performed to confirm the diagnosis. Among 19 patients, 15 had histopathologically identified cancer in peripheral zone and 4 in the central gland. Two image modalities were acquired from the pelvis of all patients using a 3.0 T MR scanner (Ingenia, Philips Medical System, Best, the Netherlands) using small field of view as follows: Axial T2W Images (T2WI) acquired with Fast-Spin-Echo (TE/TR: 4389/110 ms, Flip Angle: 90° with image resolution of 0.42 × 0.42 × 2.4 mm^3^) and axial Diffusion Weighted Images (DWI) with two *b*-values [TE/TR:4000/85 ms, FA:90°, 1.79 × 1.79 × 0.56 mm^3^, *b*-values:0 and 1000 (sec-mm^−2^)]. The voxel-wise Apparent Diffusion Coefficient (ADC) map was constructed using two DWIs with two *b*-values. A large field of view transverse T2W sequences was also acquired to access the pelvic bones and lymph nodes. Image registration and lesion contouring was performed on in-house developed software.

### Data Contouring and Harmonization

For each patient of group B, a radiologist with over 20 years of experience evaluated the axial T2WI and ADC maps and used the following criteria for delineation of DIL: Areas with a well-circumscribed, hypo-intense with the highest Gleason score in the prostate on T2WI and ADC map. DIL and the equivalent region in contralateral (normal prostatic glandular tissues, NT) were contoured on axial T2WI and ADC maps, respectively. To harmonize the data and make them independent from MR scanner gains (can affect weighted images), for each patient of both groups, the signal intensity of their DIL was normalized to the mean value of their corresponding normal volume prior to the radiomics analysis.

### Radiomics Analysis

All data processing was performed off-line using a commercial software package (MATLAB 2016a, the MathWorks Inc., Natick, MA, 2000). For each patient, 168 radiomics features (15), from eight different categories, were extracted from DIL and NT volumes contoured on ADC maps and T2W images. The 8 feature categories (15), as detailed below and in Table 2, were classified as follows: Intensity Based Histogram Features (IBHF−9 features), Gray Level Run Length (GLRL−7 features), Law's Textural information (LAWS−18 features), Discrete Orthonormal Stockwell Transform (DOST−18 features), Local Binary Pattern (LBP−6 features), Two-Dimensional Wavelet Transform (2DWT−48 features), Two Dimensional Gabor Filter (2DGF−40 features), and Gray Level Co-Occurrence Matrix (GLCM−22 features) (15).

**Table 2:** Eight different radiomics feature categories along with a short explanation of each category is shown in this table.

**Category**	**Number of radiomics features**	**Radiomics features**
IBHF	9 features	Nine features are extracted from histogram of the pixel intensity values: 1-Mean, 2-Standard Deviation, 3-Skewness, 4-Kurtosis, 5-Entropy, 6-Central Moment of 3rd order, 7- Central Moment of 4th Order, 8- Central Moment of 5th Order, 9- Central Moment of 6th Order.
GLRL	7 features	Seven Gray Level Run Length texture descriptors were constructed based on the following emphasizes: Short Run Emphasis (SRE), Long Run Emphasis (LRE), Gray Level Non-Uniformity (GLN), Run Percentage (RP), Run Length Non-Uniformity (RLN), Low Gray Level Run Emphasis (LGRE), and High Gray Level Run Emphasis (HGRE).
LAWS	18 features	Nine textural maps were constructed by filtering the image data using the following convolution kernels: L5 = [1 4 6 4 1], E5 = [−1 −2 0 2 1], S5 = [−1 0 2 0 −1], R5 = [1 −4 6 −4 1] and then, 18 LAWS textural features were computed by applying and combining the energy and entropy operators on these maps as following: L5E5/E5L5, L5R5/R5L5, E5S5/S5E5, S5S5, R5R5, L5S5/S5L5, E5E5, E5R5/R5E5, and S5R5/R5S5.
DOST	18 features	The two-dimensional matrix of DOST coefficients was divided into nine equal segments and the energy and entropy of each segment was averaged over the tumor volume and eighteen features (nine energy along with nine entropy) were generated and used as the DOST radiomics features.
LBP	6 features	Local Binary Pattern algorithm with a radial filter (eight-neighborhood) was used to generate a two-dimensional LBP map and Entropy, Entropy, Mean, Standard Deviation, Skewness, and Kurtosis of the LBP maps were used as the six LBPF radiomics features.
2DWT	48 features	Two-dimensional Wavelet Transform with six decomposition levels for four different information attributes (Multi-resolution image, vertical, horizontal, and diagonal) was used to generate 24 maps of 2DWT information. Energy and entropy of the information maps were calculated and used as the 48 2DWT radiomics features.
2DGF	40 features	Two-dimensional Gabor (2DG) filter with five different scales for four different orientations generated 20 maps. Energy and entropy of the maps was averaged over the tumor volume and used as the 2DGT radiomics features.
GLCM	22 features	Gray-Level-Co-occurrence Matrix (GLCM) was generated and the following 22 features were measured from the GLCM using an 8-bit depth quantization: 1-Autocorrelation, 2-Contrast, 3-Correlation (2), 4-Correlation (1), 5-Cluster Prominence, 6-Cluster Shade, 7-Dissimilarity, 8-Energy, 9-Entropy, 10-Homogeneity (1), 11-Homogeneity (2), 12-Maximum probability, 13-Sum of squares(Variance), 14-Sum average, 15-Sum variance, 16-Sum entropy, 17-Difference variance, 18-Difference entropy, 19-Information measure of correlation (1), 20-Information measure of correlation (2), 21-Inverse difference normalized, and 22-Inverse difference moment normalized.

### Feature Selection and Statistical Analysis

**Figure 1:**
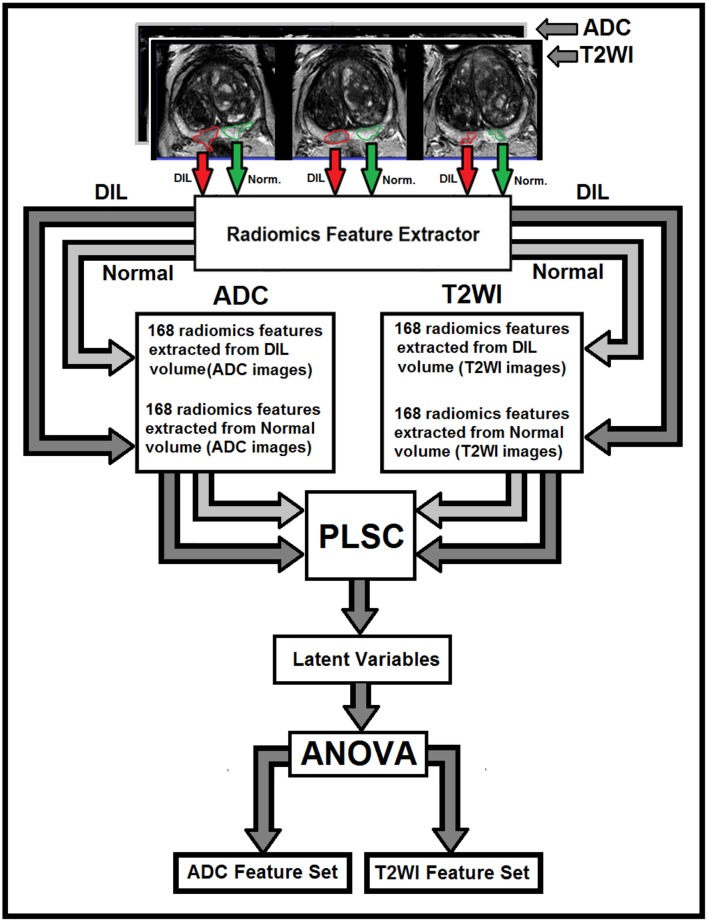
The flowchart demonstrates different steps for the extraction of radiomics features from T2W images and ADC maps for DILs and normal tissues. As shown in this figure, for each MR modality, 168 radiomics features are extracted from normal and DIL volumes. The optimal feature set for the two MR modalities are identified using ANOVA applied on the latent variables generated by the PLSC technique for features with Silhouette coefficient of 0.5 and greater.

A Partial Least Square Correlation (PLSC) (16) technique combined with one-way analysis of variance (ANOVA) were recruited to identify the most discriminant PLSC latent variables constructed from radiomics features extracted from NTs and DILs of multimodal MR information (T2WI and ADC map). PLSC method which is also called as projection to latent structures, can relate the information present in two MR modalities in which collect measurements on the same set of observations (16, 17). The goal of the PLSC is to find pairs of latent vectors with maximal covariance and with the additional constraints that the pairs of latent vectors made from two different indices are uncorrelated and the coefficients used to compute the latent variables are normalized. As shown in Figure 1, two observation matrices were constructed using 168 radiomics features extracted from the two image modalities (T2WI and ADC) from total patients. A singular value decomposition (SVD) technique was used to analyze the common and discriminant information between the two observation matrices. For each MR modality, a latent vector was computed by the SVD technique and then it was tested by the ANOVA (with homoscedasticity assumption and confidence level of 0.95) to identify the most discriminant features in latent variable space between the features extracted from DIL and NT volumes in both groups. The Holm–Bonferroni method (18) was also used for circumventing the problem of multiple comparisons for the *p*-values. This method of *p*-value adjustment controls the familywise error rate and offers a uniform test, which is more powerful than the classic Bonferroni correction (18). Using the discriminant latent variable set identified by ANOVA, an optimal feature set for both modalities was identified and constructed.

### Feature Ranking Using Bootstrapping Ratio Technique

A bootstrapping ratio (16, 19, 20) and permutation test (10,000 times randomly repeated) were performed on the latent vectors of the features sets (extracted from T2WI and ADC) and the SVD was computed for each configuration and distribution of eigen values was used to estimate the ranking and efficiency of the radiomics features against random permutation. For radiomics feature ranking, bootstrap ratios were computed by dividing the mean of the bootstrapped distribution of a significant latent variable by its standard deviation. The bootstrap ratio is akin to a Student t criterion and so if a ratio is large enough (>2.00; because it roughly corresponds to 95% of confidence level for a *t*-test) then the variable is considered significant/important for the dimension. The bootstrap estimates a sampling distribution of a statistic by computing multiple instances of this statistic from bootstrapped samples obtained by sampling with replacement from the original sample (16, 19, 20).

### Artificial Neural Networks: Architecture Optimization, Training, and Validation Strategies

Eight latent variables constructed from the radiomics information were identified as the optimal feature set and were used as the input to an artificial neural networks (ANN) with a feed-forward multilayer perceptron (MLP) architecture and back-propagation training algorithm (21) for classification of DILs and NTs. In this type of ANN, the nodes are organized in multiple layers; The ANN used in our study had three layers: the input layer, single intermediate layer, and the output layer (21, 22). Nodes were interconnected by weights in such a way that information propagates from one layer to the next, passing through a sigmoid (bipolar) activation function (22). Learning rate and momentum factors were set to control the internode weight adjustments during training (learning rate: 0.01, and Momentum: 0.01). A back propagation learning strategy (21) was employed for training the ANN in a supervised mode. In this strategy, a trial set of weights (the weight vectors, one vector for each layer of the ANN) was proposed. The initial weights were assigned randomly, and the same set of initial weights was saved and used for different trial during the leave-one-out method. The weight vectors were then adjusted to minimize some measure of error (in this case the Mean Square Error, MSE) between the output of the ANN and the training set. This procedure was performed iteratively across the entire data set using a batch processing mode to improve the convergence rate and the stability of training. The weight changes obtained from each training case were accumulated, and the weights updated after the entire set of training cases was evaluated. Batch processing improves stability, but with a tradeoff in reduction of the convergence (21–23).

**Figure 2:**
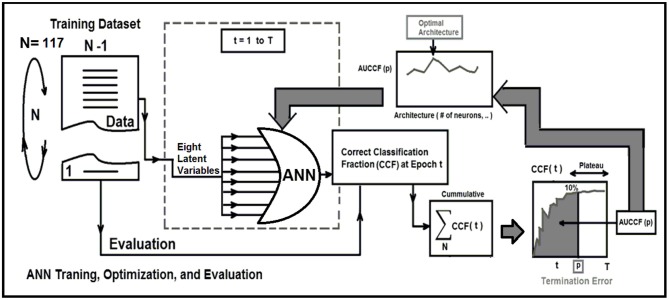
This figure demonstrate three major phases as follows: Training, optimization, and evaluation phases for the ANN using the leave-one-out technique and area under correct classification fraction.

Two different training and validation strategies were recruited and tested as follows:

*Strategy 1:* Leave-One-Out Cross-Validation (LOOCV) method, which is a particular case of the Leave-P-Out Cross Validation (called as Exhaustive Method) was employed for training, testing, and ANN architecture optimization (21, 22, 24–26). LOOCV was recruited to find the optimal structure, termination error, and validation of the ANN. As shown in Figure 2, this approach leaves one data point out of training data, i.e., if there are N data points in the original sample then, N-1 samples are used to train the model and 1 point is used as the validation. This is repeated for all combinations in which original sample can be separated this way, and then the error is averaged for all trials, to give overall effectiveness with less estimated bias (27). This method is generally preferred over the Leave-P-Out Cross Validation when the sample size is small since it does not suffer from the intensive computation, as number of possible combinations is equal to number of data points in original sample or *N* (28). Finally, to evaluate the stability of the optimal ANN against optimal number of training epochs, a series of ROC curves were generated by applying a threshold at the output of the randomly (100 times) trained ANN. The, the optimal cut-point which is the point closest-to- corner in the ROC plane was calculated. The optimal cut-point defines as the point minimizing the Euclidean distance between the ROC curve and the (0, 1) point (29). As the sensitivity (true positives) increases, the ANN can identify more cases with DIL, while the accuracy on identifying NTs (specificity) are sacrificed. Cut-points dichotomize the test values, so this provides the classification (DIL or not). Simultaneous assessment of sensitivity and specificity is used to estimate the cut-point value which is considered as optimal when the point classifies most of the individuals correctly (29, 30).

To measure how accurately the ANN matched the whole input dataset with the entire identifier set, the ANN's Correct-Classification-Fraction (CCF: True Positive plus True Negative, TP+TN) curve was generated at different levels of epochs during the LOOCV procedure. The area under Receiver-operating characteristic (AUROC, *Az-value*) curves (21, 22, 24, 25) for the ANN that is an index of predictive performance, was used to compare the ANN's performance in determining the optimal architecture of the ANN, and also finding the termination error (avoid overfitting) for training the optimal ANN.

*Strategy 2:* For each discriminant latent variable, the data of the patient group *A* (96 patients) was split 100 times into training and validation components. In each data split, two-thirds (67%) of the entire dataset was randomly sampled and used as a training set and the remaining one-third (33%) was used as the unseen cohort or validation dataset (31). Using the training and validation sets for each of the 100 iterations, the ANN was trained and validated separately for each discriminant latent variable. The same procedure was repeated for the set of eight latent variables. The AUROC, Positive Predictive value (PPV) and Negative Predictive value (NPV) were computed for each trial and were averaged to evaluate ANN classification performance for each discriminant latent variable and the set of eight latent variables.

All data processing and classifier implementation were performed using a series of in-house codes developed in the MATLAB environment.

### Testing of Data Harmonization, Feature Consistency, and Generalization Error

Data harmonization refers to all efforts to combine different datasets collected by different scanners in different institutions. Finally, in order to test the consistency of the identified discriminant latent variables against the data harmonization and also testing the performance of the classifiers against prospective/unseen datasets (ANN generalization error), the following sub-analysis was conducted: An ANN was trained using the eight discriminant latent variables (constructed from radiomics information) extracted from patients information of group *A*. The trained ANN was then applied on the eight discriminant latent variables (constructed from radiomics information) extracted from patient information of group *B* (as test set or unseen patient cohorts). Ultimately, a ROC analysis was performed on the predictions of the trained ANN and AUROC, NP, and PP values for the unseen testing cohort (group *B*) were calculated.

### Contour Variability Test

Nineteen patients were randomly chosen from hundred-seventeen patients and their DIL and NT contours were modified by scaling the contours by a factor of 1.2 in all directions followed by a 1 voxel shift in all directions and their modified contours were used to repeat the radiomics and PLSC analyses and ANOVA method was used to test the sensitivity of the latent variables against contour variability.

### Tumor Probability Map

The trained ANN and a two dimension (2D) convolutional sampling method window size = 25 × 25) were combined and used to estimate DIL-NT probability map for two test cases. Dice coefficients between the DIL contours and the DIL patch estimated from the probability maps (*P*_*thr*_ > *0.001*) for the two cases were calculated and compared.

## Results

**Figure 3:**
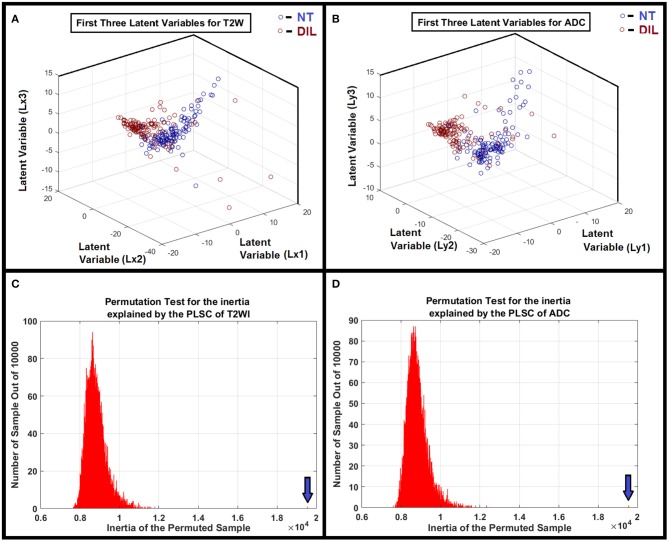
**(A,B)** Clusters of NTs and DILs for each latent variable are well-separated with less diffusivity. It confirms that the distribution of the identified latent variable (PLSC-ANOVA) in the feature space is well-matched to its own cluster (less scattered) and poorly diffused to its neighboring clusters for the MR modalities. **(C,D)** Show the results of the permutation tests for the inertia explained by the PLSC of T2WI and ADC map for 10,000 permutations. As shown in the subfigures, the observed value (shown by vertical arrows) were never obtained in the 10,000 permutations for both modalities. Therefore, it is concluded that PLSC extracted a significant amount of common variance between these two modalities with *P* < 0.0001.

A flowchart demonstrating different steps for extracting radiomics features from T2W images and ADC maps for DILs and NTs are shown in Figure 1. As shown in the figure, for each MR modality, 168 radiomics features were extracted from each of the NTs and DILs and finally, the optimal discriminant latent feature set for the two MR modalities were identified using a PLSC technique and ANOVA. Table 3 shows feature ranking results based on the PLSC and bootstrapping ratio techniques for the first 10 significant radiomic features of two MR modalities. Figures 3A,B demonstrate the scatter plots of the first three PLSC latent variables for T2WI and ADC, respectively. Figures 3C,D demonstrate the permutation tests for the inertia explained by the PLSC of the T2WI and ADC map along with their observed inertia for the 10,000 permutations.

**Table 3:** Feature ranking based on the PLSC and Bootstrapping techniques for the first 10 significant radiomic features of two MR modalities.

**Feature no**.	**Image modality**	**Radiomics feature**	**Radiomics feature category**	**Bootstrapping ratio (mean/std)**
1	T2WI	LBP_Energy	LBP	21412.01
2	ADC Map	LBP_Energy	LBP	410.83
3	T2WI	RLN	GLRL	159.69
4	ADC Map	RLN	GLRL	70.32
5	T2WI	GLN	GLRL	35.29
6	T2WI	HGRE	GLRL	35.28
7	ADC Map	DOST_ENTROPY_22	DOST	25.13
8	ADC Map	ENG_GAB02	2DGF	22.99
9	ADC Map	LBP_KURTOSIS	LBP	22.95
10	T2WI	LBP -KURTOSIS	LBP	22.92

**Figure 4:**
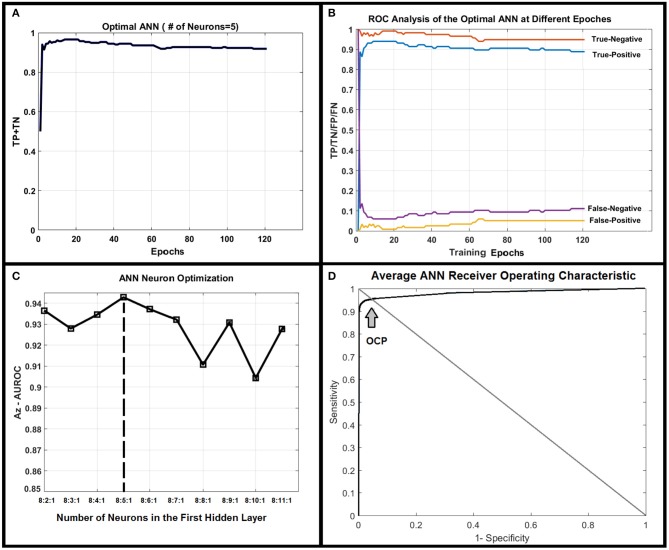
**(A)** Shows true positive plus true negative (TP+TN) of the optimal ANN (8:5:1) at different training epochs. **(B)** Shows true positive, true negative, false positive, and false negative of the optimal ANN at different training epochs. **(C)** Demonstrates the area under receiver operating characteristic (AUROC, *Az* test) value for different ANN structures. As shown in this figure, the ANN with five neurons in its only hidden layer shows the highest performance and is chosen as the optimal ANN. **(D)** Shows the average ROC of the optimal ANN along with optimal-cut-point of the ANN.

Figure 4A shows correct classification fraction (CCF = TP + TN) of the optimal ANN at different training epochs for LOOCV technique. The epoch corresponding to 10% change in plateau for the optimum architecture (8:5:1) was used as the stopping epoch (epoch = 17) of the ANN. Figure 4B shows TP, TN, false positive (FP), and false negative (FN), of the optimal ANN at different training epochs.

The AUCCF values for different ANN structures for LOOCV technique are shown in Figure 4C. As shown in this figure, the ANN with five neurons in its only hidden layer shows the highest performance (*A*_*z*_ = 0.95) and is chosen as the ANN with optimal structure. Figure 4D shows the average AUROC of the ANN generated for randomly (100 times) trained ANNs along with the optimal cut-point (OCP = 0.96). Given the average AUROC (A_z_ test ~ 0.96), the optimal cut-point of the ANN, and the eigen value distributions for the randomly permuted (10,000 permutations) radiomics features, the generalization error of the ANN was about 4% with a very strong permutation-invariant efficiency, *p* < 0.0001) against the order of the latent variables.

**Figure 5:**
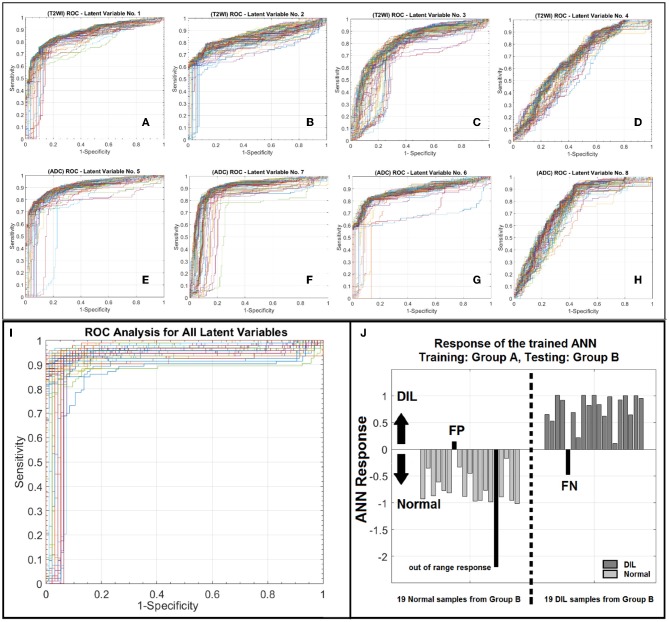
**(A–D)** Depict ROC curves corresponding to 100 iterations each corresponding to a different division of training and validation datasets for ANN for the T2WI latent variables number 1–4. **(E–H)** depict ROC curves corresponding to 100 iterations each corresponding to a different division of training and validation datasets for ANN for the ADC latent variables number 1–4. As shown in this figure for each modality, from left to right as the order of latent variable increases the information content or discrimination power of the variable for classification deceases. **(I)** illustrates a family of ROC curves for 100 iterations, each corresponding to a different division of training and validation datasets for ANN for all 8 latent variables. **(J)** shows the response of the trained ANN against an unseen/prospective dataset (trained with group A and tested with group B).

AUROC, PPV, and NPV for the conventional method were 94%, 0.95, and 0.92, respectively. ROC analyses for eight individual latent variables (4 for T2WI and 4 for ADC) are shown in Figure 5. Figures 5A–D demonstrate the ROC analyses of the ANN for the first 4 latent variables constructed from T2WI for 100 random iteration corresponding to a different division of training and validation data of group *A* while Figures 5E–H depict the corresponding information for the ADC map. Table 4 shows AUROC, NPV, and PPV values along with their confidence intervals measured for each individual latent variable for 100 iterations (each corresponding to a different division of training and validation datasets).

**Table 4:** This table shows AUROC, NPV, and PPV values along with their confidence intervals measured for each individual latent variable for 100 iterations (each corresponding to a different division of training and validation datasets).

**Latent variable**	**AUROC**	**AUROC-CI**	**PPV**	**PPV-CI**	**NPV**	**NPV-CI**
First latent variable (T2WI)	0.87	0.86–0.89	0.86	0.84–0.88	0.78	0.76–0.79
Second latent variable (T2WI)	0.79	0.72–0.85	0.84	0.78–0.90	0.71	0.68–0.74
Third latent variable (T2WI)	0.76	0.75–0.80	0.72	0.67 0.74	0.72	0.70–0.74
Fourth latent variable (T2WI)	0.66	0.64–0.68	0.58	0.57–0.60	0.69	0.65–0.73
First latent variable (ADC)	0.91	0.90–0.92	0.88	0.85–0.90	0.82	0.80–0.84
Second latent variable (ADC)	0.88	0.86–0.89	0.87	0.85–0.89	0.81	0.80–0.83
Third latent variable (ADC)	0.79	0.72–0.87	0.80	0.75–0.85	0.83	0.81–0.86
Fourth latent variable (ADC)	0.74	0.72–0.76	0.66	0.64–0.67	0.81	0.78–0.85

**Figure 6:**
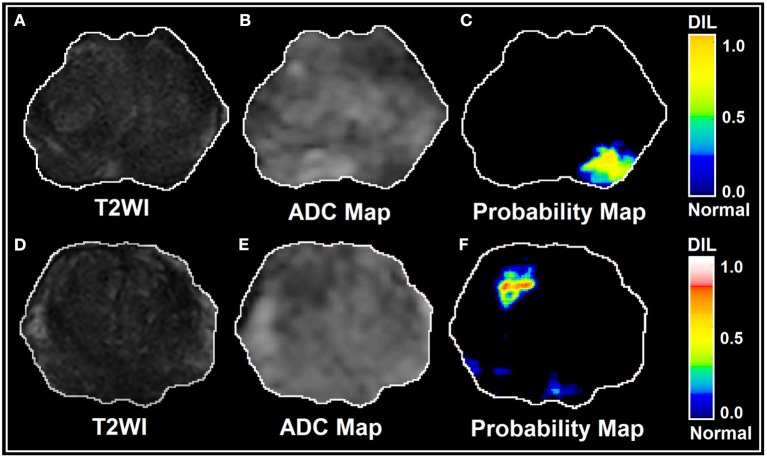
**(A–F)** illustrate T2WI, ADC map, and lesion probability map for a slice of prostate gland for two different patients estimated by the trained PLSC-ANN using a 2D-convolutional sampling method (window size = 25 × 25).

As shown in Figure 5I, for the conventional training and validation method, the average AUROC, PPV and NPV were 95%, 0.96, and 0.93, respectively. Figure 5J shows the response of the trained ANN (group *A*) when it was applied on group B. The performance of the trained ANN (using group *A* dataset) when it was applied on the unseen data cohort (group *B*) was: Sensitivity/Specificity = 0.95/0.94. The radiomic-based latent variables of the NTs and DILs showed no statistically significant differences (F_statistic_ for all 8 latent variables were smaller than F_critical_ = 4.11, with Confidence Level of 95%) against contour variability. Figures 6A–F, illustrate T2WI, ADC map, and lesion probability map for a slice of prostate gland of two different patients estimated by the trained ANN using a 2D-convolutional sampling method (window size = 25 × 25). Dice coefficients between DIL-NT probability map and physician contours for the two test cases were 0.82 and 0.71, respectively.

## Discussion

Recent studies have shown that cancerous tissues are spatially heterogeneous due to factors, such as cell structures, genes, protein contents, cell morphologies, tumor microenvironment, and physiology (32). Indeed, the main purpose of using radiomics is to reveal and extract additional information from medical imaging modalities, associated with *macroscopic* and *microscopic* image-based features that have the potential to serve as surrogates for pathophysiological and radiological parameters, such as tumor heterogeneity level, pathology, response to a given therapy, decoration and distribution of information in images, and structural and image-based patterns in digital images. In our study, given the variation and nature of the radiomics features, we extracted multi scale information in form of features from the prostate gland to characterize normal prostatic tissue and tumor phenotypes from multi model MRI.

The PLSC technique used in this study allowed the finding of shared information between the two image modalities (T2WI and ADC). This approach is equivalent to a correlation problem (16, 17, 33) and provided descriptive features from multivariate information in form of latent variables which are optimal linear combinations of the variables extracted from the two image modalities. Partial least square (PLS) method that benefits from projecting feature information on latent structures, relates the information present in two data tables (modalities) that collect measurements on the same set of observations (16). PLSC latent variables constructed on the basis of radiomics information extracted from DIL and NT consists of all radiomics features and can help reveal variations of descriptive features or discriminant parameters for classification of DIL from NT. An adaptive classifier (such as ANN) provides capability of implicitly detecting complex non-linear relationships between dependent and independent radiomics variables (already found as optimal feature set in latent variable space) and their variations, modeling their non-linear changes as well as detecting all possible interactions between the predictor variables. As shown in Figures 3A,B, clusters of NTs and DILs for each latent variable are well-separated with less diffused marginal points in the feature space. It confirms that the distribution of the identified latent variable (PLSC-ANOVA) in the PLSC space is well-matched to its own cluster (less scattered) and poorly diffused to its neighboring clusters. Figures 3C,D show the results of the permutation tests for the inertia explained by the PLSC of T2WI and ADC map for 10,000 permutations. The observed value (shown by vertical arrows) were never obtained in the 10,000 permutations for both modalities. Therefore, it is concluded that PLSC technique was able to successfully extract significant amount of common variances between these two modalities with *p*-value smaller than 0.0001.

Recruitment of PLSC technique and ANOVA in this study allowed robust comparison and revealing of the correlation and descriptive power of different radiomics features extracted from the two MR modalities, while providing more predictive accuracy and a much lower chance of risk for the two sets of features affecting each other. The major limitations could be the sensitivity to the relative scaling of the descriptor variables that was addressed by the standardization and harmonization steps prior to the feature extraction.

Recent studies (34–39) have shown that ADC measurements are affected by the user selected repetition time (T_R_) values, especially if it is comparable to the relaxation time. The degree of T_R_ dependence is also codependent on another parameter called number of diffusion preparation pulses. Similar to T_R_ dependence of ADC values, it is expected that there could be an echo time (T_E_) dependence on ADC values. In fact, Wang et al. (39) found a modest correlation between T_E_ and ADC values in the prostate. It has been shown that tissue specific relaxation time parameters such as T1 and T2 and imaging parameters such as T_R_ and T_E_ affects the optimum b-value for different anatomies, tissues, and even lesion types within the same organ. Therefore, since the ADC value could be highly and “*non-linearly*” affected by the MR imaging parameters (34–39), in this study, as part of data harmonization, normalization to normal volume was performed to suppress the effect of the MR imaging parameters on the ADC values. Such normalization made the ANN independent and less sensitive to the MR imaging parameters for prospective patients whom could be scanned with different scanners or different imaging parameters.

As shown in Figure 5 and according to the statistical measures reported in Table 4, as it is expected, for each modality, from left to right (Figures 5A–D or Figures 5E–H), as the order of the latent variable increases the information content or discrimination power of the variable for DIL classification deceases. As shown in Table 4 and Figure 5, the analysis results strongly confirm that compared to T2WI modality, the ADC modality is more discriminative with higher information content for the classification of DILs and NTs.

The application of novel machine learning techniques such as Bayesian approach, Support vector machine (SVM) kernels: polynomial, radial base function (RBF) and Gaussian and Decision Tree for detecting prostate cancer have been proposed by several research groups (40–42). Moreover, different features extracting strategies are proposed to improve the DIL detection performance (40). ANNs have been used in different fields on a variety of tasks such as computer vision, speech recognition, machine translation, social network filtering, medical diagnosis, and in many other domains. There have been numerous applications of ANNs within medical decision-making (26, 43, 44). It has been shown that ANNs have unique properties including robust performance in dealing with noisy or incomplete input patterns, high fault tolerance, and the ability to generalize from the training data (26, 43). The adaptive model constructed in this study can benefit from the ANN's properties stated above and can distinguish DILs from NTs with almost uniform sensitivity at different levels of specificities (see Figures 4A,B, 5I). The stability (lesions being non-patchy and uniform) of the predicted DILs and NTs in the probability maps (shown in Figure 6) clearly confirm the robustness of the PLSC-ANN technique in information extraction from the two MR modalities. The proposed ANN in this study was trained without any data augmentation. The results implied that the trained ANN can also evaluate any suspicious lesion in different zones of the prostate gland (PZ or TZ) regardless of its Gleason score.

Our study also confirms that the most discriminant features are textural-based features and given the bootstrapping feature ranking results, it can be concluded that frequency or arrangement-based features (LBP, GLRL, DOST, and 2DGF, see Table 3, a measure of the decoration or disorder of information distribution within a region), that are associated with subtle and descriptive information content of the two image modalities, play a key role in discrimination of DIL from NT. Also, we did not include morphological features such as volume, shape, solidity, convexity, eccentricity, and etc. in order to eliminate any possible biasing result from the manual contouring of DILs and NTs.

In this study, DIL and the NT contours were separately drawn on each image modality. While such a process could increase the chance of contour variability and negatively increase the variation of the data, it had an advantage that the two image modalities (T2W images and ADC map) did not necessarily need to be co-registered to each other prior to the radiomic analysis and adaptive modeling and therefore, the analysis results were not negatively affected by any possible co-registration errors. DILs and NTs contoured on unregistered image modalities were directly used for training and testing of the ANN. We only co-registered the two image modalities (T2WI and ADC map) using rigid co-registration [affine transform (45)] method for the two test cases (see Figure 6) to predict DIL-NT probability map using the trained ANN and 2D-convolutional sampling method.

The current major computer aided diagnosis systems recorded AUROC performance ranging from 0.77 to 0.89 and the focus was to detect lesions in the peripheral zone. Most image features, either individually or in combination that were effective in the differentiation of prostate cancer, are volume averaged quantities such as the 10th percentile of the ADC, T2W signal intensity skewness (46). Niaf et al. studied texture features extracted from MP-MRI on 30 fully annotated patients using four different feature selection and classification methods (47). They could achieve a diagnostic performance of 0.89 but the study was limited to the peripheral zone only. The performance was poorer due to the overfitting problem when all features were used for classification.

In this study, despite using 117 subjects (two cohorts: 96, and 19) with two different training and validation strategies, there are still several challenges as follows: Compared to the number of radiomics features, the study is limited by the number of patients, which will impact the optimal features selected, and also might render a predictive model susceptible to Type II errors. A larger sample size will also allow the construction of a more reliable ANN in order to draw a reliable and unequivocal conclusion.

In this study, two different training and validation strategies were recruited and the strong agreement between the analysis results confirmed the robustness of the identified features. In the first strategy, employing the LOOCV method in this study, allowed us to use a high proportion of the available training data fraction (1–1/K = 0.99 for K = 117), for training, while making use of all the data in estimating the generalization error or agreement. The cost is that the process can be lengthy, since we need to train and evaluate the network K times. Typically, according to the literatures, K ≈ 10 is considered reasonable (48). In this study, K was set to 117 for 117 patients (one case with DIL and NT in each fold) and the ANN had a single output, to predict the outcome. The radiomics features selected might be impacted by the intensities, size of the contour, and contrast of the NT. Since the region of interests were delineated manually, the accuracy and variability of the ROIs could impact on the optimal feature selection and the training results.

The Az-test for the average ROC analysis of the ANN is 1% higher than the Az-test of the optimal ANN (see Figures 3C,D). This is due to the difference between the way the two tests are conducted: for average AUROC, each NT or DIL from each subject is considered as a sample (thus the total samples are equal to 234) while in the ordinary Az-test for the optimal ANN, pair of NT and DIL for each subject is considered as a sample (thus the total samples are equal to 117). Strong agreement between the statistical measures of the LOOCV and conventional methods and also the high predictive power of the trained ANN (group A) when it was applied on group B (as prospective or unseen data cohort), confirm the consistency and high information content of the discriminant features identified in this study.

The 2D-convolutional sampling analysis results presented in Figure 6, imply that the trained-ANN is capable of estimating the DIL and normal tissue probabilities when the target contour (the 2D window) consists of a mixed radiomic information extracted from DIL and normal tissue.

ANN was implanted as a classifier since it has high tolerance against variation of input feature components and contours (according to the contour variability test results) while they are less sensitive to random noise (49), which allows the construction of a variation- and noise-insensitive adaptive classifier with higher accuracy and speed. Most importantly, ANN considers non-linear relationships among input data that cannot always be recognized by conventional analyses. Results of the permutation test also imply that the discriminant features used for training, are reliable and efficient for classification.

## Conclusion

In conclusion, this study demonstrates the high performance of combining radiomics analysis, PLSC technique and adaptive model for extracting and ranking features from multimodal MR information such as T2WI and ADC maps to detect DILs and NTs in patients with PCa. The radiomics information of ADC modality was proved to have higher discrimination power compared to the corresponding features extracted from T2WI modality. Results are suggestive that the integration of quantitative image analysis methods such as radiomics analysis and PLSC technique when combined with an adaptive model can help identify imaging biomarkers and show great potential to help clinicians improve the classification of clinically significant prostate lesions for therapy of prostate cancer.

## Data Availability Statement

The raw data supporting the conclusions of this manuscript will be made available by the authors, without undue reservation, to any qualified researcher.

## Ethics Statement

The studies involving human participants were reviewed and approved by The Internal Review Board at Henry Ford Health System. Written informed consent for participation was not required for this study in accordance with the national legislation and the institutional requirements.

## Author Contributions

HB-E and NW designed the research and methodology. HB-E, CL, BJ, and NW performed the research. HB-E and CL contributed to the data pre-processing. HB-E developed statistical analysis, PLSC, ANN training, and validation as well as the development of analytical tools. HB-E and NW wrote the paper. MP and BJ investigated the data and also contoured and labeled the tumors and normal tissues on the MR images using the pathology images. DH helped with the implementation of the MR pulse sequences. HB-E, ME, BM, IC, and NW advised and mentored the study.

### Conflict of Interest

The authors declare that the research was conducted in the absence of any commercial or financial relationships that could be construed as a potential conflict of interest.

## References

[1] Dinh CV Steenbergen P Ghobadi G Heijmink SW Pos FJ Haustermans K. Magnetic resonance imaging for prostate cancer radiotherapy. Phys Med. (2016) 32:446–51. 10.1016/j.ejmp.2016.01.484 26858164

[2] Moghanaki D Turkbey B Vapiwala N Ehdaie B Frank SJ McLaughlin PW. Advances in prostate cancer magnetic resonance imaging and positron emission tomography-computed tomography for staging and radiotherapy treatment planning. Semin Radiat Oncol. (2017) 27:21–33. 10.1016/j.semradonc.2016.08.008 27986208 PMC5743235

[3] Dearnaley DP Jovic G Syndikus I Khoo V Cowan RA Graham JD. Escalated-dose versus control-dose conformal radiotherapy for prostate cancer: long-term results from the mrc rt01 randomised controlled trial. Lancet Oncol. (2014) 15:464–73. 10.1016/S1470-2045(14)70040-3 24581940

[4] Li B Du Y Yang H Huang Y Meng J Xiao D. Magnetic resonance imaging for prostate cancer clinical application. Chin J Cancer Res. (2013) 25:240–9. 10.3978/j.issn.1000-9604.2013.03.06 23592906 PMC3626986

[5] Anderson ES Margolis DJ Mesko S Banerjee R Wang PC Demanes DJ. Multiparametric MRI identifies and stratifies prostate cancer lesions: implications for targeting intraprostatic targets. Brachytherapy. (2014) 13:292–8. 10.1016/j.brachy.2014.01.011 24709516

[6] Rischke HC Nestle U Fechter T Doll C Volegova-Neher N Henne K. 3 Tesla multiparametric MRI for gtv-definition of dominant intraprostatic lesions in patients with prostate cancer–an interobserver variability study. Radiat Oncol. (2013) 8:183. 10.1186/1748-717X-8-183 23875672 PMC3828667

[7] Turkbey B Mani H Shah V Rastinehad AR Bernardo M Pohida T. Multiparametric 3T prostate magnetic resonance imaging to detect cancer: histopathological correlation using prostatectomy specimens processed in customized magnetic resonance imaging based molds. J Urol. (2011) 186:1818–24. 10.1016/j.juro.2011.07.013 21944089 PMC5540658

[8] Delongchamps NB Beuvon F Eiss D Flam T Muradyan N Zerbib M. Multiparametric MRI is helpful to predict tumor focality, stage, and size in patients diagnosed with unilateral low-risk prostate cancer. Prostate Cancer Prostatic Dis. (2011) 14:232–7. 10.1038/pcan.2011.9 21423266

[9] Kumar V Gu Y Basu S Berglund A Eschrich SA Schabath MB. Radiomics: the process and the challenges. Magn Reson Imaging. (2012) 30:1234–48. 10.1016/j.mri.2012.06.010 22898692 PMC3563280

[10] Aerts HJ Velazquez ER Leijenaar RT Parmar C Grossmann P Carvalho S. Decoding tumour phenotype by noninvasive imaging using a quantitative radiomics approach. Nat Commun. (2014) 5:4006. 10.1038/ncomms5644 24892406 PMC4059926

[11] Litjens G Debats O Barentsz J Karssemeijer N Huisman H. Computer-aided detection of prostate cancer in MRI. IEEE Trans Med Imaging. (2014) 33:1083–92. 10.1109/TMI.2014.2303821 24770913

[12] Fei B. Computer-aided diagnosis of prostate cancer with MRI. Curr Opin Biomed Eng. (2017) 3:20–7. 10.1016/j.cobme.2017.09.009 29732440 PMC5931723

[13] Lemaître G Martí R Freixenet J Vilanova JC Walker PM Meriaudeau F. Computer-aided detection and diagnosis for prostate cancer based on mono and multi-parametric MRI: a review. Comput Biol Med. (2015) 60:8–31. 10.1016/j.compbiomed.2015.02.009 25747341

[14] Barentsz JO Richenberg J Clements R Choyke P Verma S Villeirs G. ESUR prostate mr guidelines 2012. Eur Radiol. (2012) 22:746–57. 10.1007/s00330-011-2377-y 22322308 PMC3297750

[15] Bagher-Ebadian H Siddiqui F Liu C Movsas B Chetty IJ. On the impact of smoothing and noise on robustness of CT and CBCT radiomics features for patients with head and neck cancers. Med Phys. (2017) 44:1755–70. 10.1002/mp.12188 28261818

[16] Abdi H Williams LJ. Partial least squares methods: partial least squares correlation and partial least square regression. Methods Mol Biol. (2013) 930:549–79. 10.1007/978-1-62703-059-5_23 23086857

[17] Grellmann C Bitzer S Neumann J Westlye LT Andreassen OA Villringer A. Comparison of variants of canonical correlation analysis and partial least squares for combined analysis of MRI and genetic data. Neuroimage. (2015) 107:289–310. 10.1016/j.neuroimage.2014.12.025 25527238

[18] Holm S A simple sequentially rejective multiple test procedure. Scand J Stat. (1979) 6:65–70.

[19] Abdi H Dunlop JP Williams LJ. How to compute reliability estimates and display confidence and tolerance intervals for pattern classifiers using the bootstrap and 3-way multidimensional scaling (distatis). Neuroimage. (2009) 45:89–95. 10.1016/j.neuroimage.2008.11.008 19084072

[20] McIntosh AR Chau WK Protzner AB. Spatiotemporal analysis of event-related fmri data using partial least squares. Neuroimage. (2004) 23:764–75. 10.1016/j.neuroimage.2004.05.018 15488426

[21] Freeman JA Skapura DM Neural Networks: Algorithms, Applications, and Programming Techniques. Reading, MA: Addison-Wesley (1991).

[22] Looney CG Pattern Recognition Using Neural Networks : Theory and Algorithms for Engineers and Scientists. New York, NY: Oxford University Press (1997).

[23] Begg R Kamruzzaman J Sarkar R Neural Networks in Healthcare: Potential and Challenges. Hershey, PA: Idea Group Pub (2006).

[24] Bagher-Ebadian H Jafari-Khouzani K Mitsias PD Lu M Soltanian-Zadeh H Chopp M. Predicting final extent of ischemic infarction using artificial neural network analysis of multi-parametric mri in patients with stroke. PLoS ONE. (2011) 6:e22626. 10.1371/journal.pone.0022626 21853039 PMC3154199

[25] Bagher-Ebadian H Nagaraja TN Paudyal R Whitton P Panda S Fenstermacher JD. MRI estimation of contrast agent concentration in tissue using a neural network approach. Magn Reson Med. (2007) 58:290–7. 10.1002/mrm.21332 17654573

[26] Pasini A. Artificial neural networks for small dataset analysis. J Thorac Dis. (2015) 7:953–60. 10.3978/j.issn.2072-1439.2015.04.61 26101654 PMC4454870

[27] Beau-Faller M Degeorges A Rolland E Mounawar M Antoine M Poulot V. Cross-validation study for epidermal growth factor receptor and KRAS mutation detection in 74 blinded non-small cell lung carcinoma samples: a total of 5550 exons sequenced by 15 molecular French laboratories (evaluation of the EGFR mutation status for the administration of EGFR-TKIs in non-small cell lung carcinoma [ERMETIC] project–part 1). J Thorac Oncol. (2011) 6:1006–15. 10.1097/JTO.0b013e318211dcee 21532509

[28] Kohavi R A study of cross-validation and bootstrap for accuracy estimation and model selection. In: Proceedings of the Fourteenth International Joint Conference on Artificial Intelligence. San Mateo, CA: Morgan Kaufmann (1995).

[29] Perkins NJ Schisterman EF. The inconsistency of “optimal” cutpoints obtained using two criteria based on the receiver operating characteristic curve. Am J Epidemiol. (2006) 163:670–5. 10.1093/aje/kwj063 16410346 PMC1444894

[30] Unal I. Defining an optimal cut-point value in roc analysis: an alternative approach. Comput Math Methods Med. (2017) 2017:3762651. 10.1155/2017/3762651 28642804 PMC5470053

[31] Steyerberg EW Harrell FE Jr. Prediction models need appropriate internal, internal-external, and external validation. J Clin Epidemiol. (2016) 69:245–7. 10.1016/j.jclinepi.2015.04.005 25981519 PMC5578404

[32] Gevaert O Xu J Hoang CD Leung AN Xu Y Quon A. Non-small cell lung cancer: identifying prognostic imaging biomarkers by leveraging public gene expression microarray data–methods and preliminary results. Radiology. (2012) 264:387–96. 10.1148/radiol.12111607 22723499 PMC3401348

[33] Ziegler G Dahnke R Winkler AD Gaser C. Partial least squares correlation of multivariate cognitive abilities and local brain structure in children and adolescents. Neuroimage. (2013) 82:284–94. 10.1016/j.neuroimage.2013.05.088 23727321

[34] Celik A. Effect of imaging parameters on the accuracy of apparent diffusion coefficient and optimization strategies. Diagn Interv Radiol. (2016) 22:101–7. 10.5152/dir.2015.14440 26573977 PMC4712890

[35] Ogura A Hayakawa K Miyati T Maeda F. Imaging parameter effects in apparent diffusion coefficient determination of magnetic resonance imaging. Eur J Radiol. (2011) 77:185–8. 10.1016/j.ejrad.2009.06.031 19646836

[36] Qin W Yu CS Zhang F Du XY Jiang H Yan YX. Effects of echo time on diffusion quantification of brain white matter at 1.5 T and 3.0 T. Magn Reson Med. (2009) 61:755–60. 10.1002/mrm.21920 19191286

[37] Saritas EU Lee JH Nishimura DG. Snr dependence of optimal parameters for apparent diffusion coefficient measurements. IEEE Trans Med Imaging. (2011) 30:424–37. 10.1109/TMI.2010.2084583 20934948

[38] Soman S Holdsworth SJ Skare S Andre JB Van AT Aksoy M. Effect of number of acquisitions in diffusion tensor imaging of the pediatric brain: optimizing scan time and diagnostic experience. J Neuroimaging. (2015) 25:296–302. 10.1111/jon.12093 24593174

[39] Wang S Peng Y Medved M Yousuf AN Ivancevic MK Karademir I. Hybrid multidimensional T(2) and diffusion-weighted mri for prostate cancer detection. J Magn Reson Imaging. (2014) 39:781–8. 10.1002/jmri.24212 23908146 PMC4251798

[40] Hussain L Ahmed A Saeed S Rathore S Awan IA Shah SA. Prostate cancer detection using machine learning techniques by employing combination of features extracting strategies. Cancer Biomark. (2018) 21:393–413. 10.3233/CBM-170643 29226857 PMC13078284

[41] Lv D Guo X Wang X Zhang J Fang J. Computerized characterization of prostate cancer by fractal analysis in MR images. J Magn Reson Imaging. (2009) 30:161–8. 10.1002/jmri.21819 19557732

[42] Tiwari P Kurhanewicz J Madabhushi A. Multi-kernel graph embedding for detection, gleason grading of prostate cancer via MRI/MRS. Med Image Anal. (2013) 17:219–35. 10.1016/j.media.2012.10.004 23294985 PMC3708492

[43] Hu X Cammann H Meyer HA Miller K Jung K Stephan C. Artificial neural networks and prostate cancer–tools for diagnosis and management. Nat Rev Urol. (2013) 10:174–82. 10.1038/nrurol.2013.9 23399728

[44] Schwarzer G Schumacher M. Artificial neural networks for diagnosis and prognosis in prostate cancer. Semin Urol Oncol. (2002) 20:89–95. 10.1053/suro.2002.32492 12012294

[45] Berger M Geometry. Berlin: Springer (1977).

[46] Peng Y Jiang Y Yang C Brown JB Antic T Sethi I. Quantitative analysis of multiparametric prostate MR images: differentiation between prostate cancer and normal tissue and correlation with gleason score–a computer-aided diagnosis development study. Radiology. (2013) 267:787–96. 10.1148/radiol.13121454 23392430 PMC6940008

[47] Niaf E Rouvière O Mège-Lechevallier F Bratan F Lartizien C. Computer-aided diagnosis of prostate cancer in the peripheral zone using multiparametric MRI. Phys Med Biol. (2012) 57:3833–51. 10.1088/0031-9155/57/12/3833 22640958

[48] Bishop CM Neural Networks for Pattern Recognition. London: Oxford University Press (1997).

[49] Miloš J Madi VJM. Assessing the sensitivity of the artificial neural network to experimental noise: a case study. FME Trans. (2010) 38:189–195. Available online at: https://pdfs.semanticscholar.org/985a/96ee91f2418baf32544500eb7e7ddaf0077d.pdf?_ga=2.269201969.1397016782.1574085333-336839267.1574085333

